# COVID-19 Vaccination of HCWs in the First Phase of a Large-Scale Mass Vaccination Program within a Healthcare System

**DOI:** 10.1017/ash.2021.20

**Published:** 2021-07-29

**Authors:** Kimberly Korwek, E. Jackie Blanchard, Julia Moody, Katherine Lange, Ryan Sledge, Maria Benedetti, Kenneth Sands

## Abstract

**Background:** The approval of the first SARS-COV-2 vaccines for COVID-19 were accompanied by unprecedented efforts to provide vaccination to healthcare workers and first responders. More information about vaccine uptake in this group is needed to better refine and target educational messaging. **Methods:** HCA Healthcare used federal guidance and internal experience to create a systemwide mass vaccination strategy. A closed point-of-dispensing (POD) model was developed and implemented. The previously developed enterprise-wide emergency operations strategy was adapted and implemented, which allowed for rapid development of communications and operational processes. A tiering strategy based on recommendations from the National Academies was used in conjunction with human resources data to determine vaccine eligibility for the first phase of vaccination. A comprehensive data and reporting strategy was built to connect human resources and vaccine consent data for tracking vaccination rates across the system. **Results:** Vaccination of employed and affiliated colleagues began December 15, 2020, and was made available based on state-level release of tiers. Within the first 6 weeks, in total, 203,544 individuals were eligible for vaccine based on these criteria. Of these, 181,282 (89.1%) consented to and received vaccine, 19,788 (9.7%) declined, and 2,474 (1.2%) indicated that they had already been vaccinated. Of those eligible, the highest acceptance of vaccine was among the job codes of specialists and professionals (n = 7,914 total, 100% consent), providers (n = 23,335, 99.6%,), and physicians (n = 3,218, 98.4%). Vaccine was most likely to be declined among job codes of clerical and other administrative (n = 12,889 total, 80.1% consent), clinical specialists and professionals (n = 22,853, 81.0%,) and aides, orderlies and technicians (n = 17,803, 82.6%,). Registered nurses made up the largest eligible population (n = 56,793), and 89.5% of those eligible consented to receive vaccination. Average age among those who consented was slightly older (48.3 years) than those that declined (44.7 years), as was length of employment tenure (6.9 vs 5.0 years). **Conclusion:** A large-scale, closed POD, mass vaccination program was able to vaccinate nearly 200,000 healthcare workers for SARS-CoV-2 in 6 weeks. This program was implemented in acute-care sites across 20 different US states, and it was able to meet the various state-level requirements for management of processes, product, and required reporting. The development of a standardized strategy and custom, centralized monitoring and reporting facilitated insight into the characteristics of early vaccine adopters versus those who decline vaccination. These data can aid in the refining and targeting of educational materials and messaging about the SARS-CoV-2 vaccine.

**Funding:** No

**Disclosures:** None

